# Research Note: The gut microbiota varies with dietary fiber levels in broilers

**DOI:** 10.1016/j.psj.2022.101922

**Published:** 2022-04-21

**Authors:** Mohan Qiu, Junqing Hu, Han Peng, Bo Li, Jingliang Xu, Xiaoyan Song, Chunlin Yu, Zengrong Zhang, Xiaogang Du, Guixian Bu, Anqi Huang, Xingfa Han, Xianyin Zeng, Chaowu Yang, Fanli Kong

**Affiliations:** ⁎ China Animal Breeding and Genetics Key Laboratory of Sichuan Province, Sichuan Animal Science Academy, Chengdu, PR 610066 China; † College of Life Science, Sichuan Agricultural University, Ya'an 625014, PR China

**Keywords:** broiler, gut microbiota, dietary fiber, short-chain fatty acids

## Abstract

It is generally accepted the gut microbiota have a profound effect on the nutrition, health, and production in poultry. To deeply understand the gut microbiota composition with the dietary fiber level in broilers, we evaluated the cecal microbiota profiles feeding on different dietary fiber level with alfalfa as additive in Dahen broilers based on 16S rRNA gene sequencing and gas chromatography. As a result, the gut microbiota diversity was greatly accelerated with the dietary fiber level. The dietary fiber stimulated the growth of many intestinal communities such as *Rikenellaceae* RC9 gut group, *Faecalibacterium, Prevotellaceae* UCG 001 and *Ruminococcaceae* UCG 014, and led to an altered microbial function such as Carbohydrate metabolism and Genetic information processing. Meanwhile, we found the genera *Anaerofilum* and *Dielma* were significantly correlated with the production of short chain fatty acids (**SCFAs**). All these results provide a reference for the broilers gut microbiota changes with different dietary fiber level. The key role of the altered microbiota with the dietary fiber may mediate beneficial effects in broiler production, which also reflect the substantial potential of dietary fiber level in poultry.

## INTRODUCTION

Broiler chickens serve as an important source of animal protein for human, and an experimental model for the basic and applied research. In this context, the improvements in growth performances and health are the key goals for the broilers production. It has been well recognized that the chicken gut microbiota plays significant roles in host health, productivity, and disease (Clavijo and Florez, 2018). Indeed, much efforts have done into optimizing the chicken gut microbiota by dietary interventions, for example, antibiotic growth promoters (**AGPs**). However, the widespread use of antibiotics in poultry production has not only changed the intestinal micro-ecosystem, but also led to the emergence of antibiotic resistance and its potential spread to threaten humans. The European Union has banned in use AGPs since 2006, thus the development of safe alternatives become a global focus. One of the alternatives for antibiotic usage is dietary fibers with prebiotic functions, which has been received much attention for its specific changes in the composition and/or activity of the gut microbiota. Recent advances in the next generation sequencing technologies have provide a greater insight into the mechanisms of gut microbiota with different treatments in host health.

Dietary fibers (**DF**), essential for a healthy diet, can indirectly promote the digestion of nutrients with the help of gut microbiota and improve the intestinal health. The chicken ceca consist of paired blind punches that can ferment and degrade the most indigestible fiber to short-chain fatty acids (**SCFAs**), principally acetate, propionate and butyrate for host energy source (David et al., 2014), while the lack of fiber in diets can reduce the diversity of gut microbiota to affect the intestinal health in poultry. Although clear evidence has demonstrated the effect of the gut microbiota on host phenotypes, the degree to which gradient dietary fibers can modulate the microbial composition remains an open question. Yet, the effects on gut microbiota that alfalfa meal as the main supplementary fiber resource fed on chicken has not been well known.

Therefore, in this study, we performed high-throughput sequencing of the V3–V4 regions of the bacterial 16S rRNA gene to assess changes in the cecum microbiota, and SCFAs of their metabolites by gas chromatography in Dahen broilers fed on different dietary fiber levels. Our finding will provide a foundation for understanding the types of the microbial community in different dietary fibers levels and their association with promoting health in broiler breeding.

## MATERIALS AND METHODS

### Study Design

Dahen broiler, a Chinese native breed developed by Sichuan Daheng poultry breeding Co., Ltd., was selected for this study, which is characterized by excellent meat quality. The one-day-old chicks were randomly assigned to 4 groups. The four groups were as follows: the control group received a commercial and refined basic diet. The basic diet consisting mostly crude fiber (**CF**) level at 2.5%, corn germ meal as fiber resource. The treatment groups were fed the base diet plus 5% CF, 7% CF, and 9% CF, supplemented with alfalfa meal (Chengdu Quanwei feed Technology Co., Ltd., China) as main fiber resource. All chicks were hatched on the same day and reared in a poultry facility according to the Chinese chicken breeding standard (NY/t33-2004). All chicks had free access to feed and water. Average daily gain (**ADG**), and average daily feed intake (**ADFI**) were recorded. All chickens in each group were weighted individually at d 1, and then with each week until slaughter at d 75.

The animal experimental procedures were approved by the Sichuan Agricultural University Animal Care and Use Committee.

### Sampling, DNA Extraction, and Sequencing

At the age of 75 d, one randomly selected chicken individual from each repeat (30 repeats/group, 15 Female /15 Male for each repeat) was slaughtered. After the abdomen was open, the cecum was immediately removed and dissected and the luminal contents were collected. All samples were snap-frozen in liquid nitrogen, and transported to the laboratory in a dry-ice pack, then stored at −80°C for subsequent studies.

Next, a total of 120 samples were separately thawed on ice and subjected to bacterial genomic DNA isolation by using the E.Z.N.A. Stool DNA Kit (D4015-02 200 preps) according to the manufacturer's instructions (Omega Bio-tek, Inc., Norcross, GA). The V3–V4 regions of the bacterial 16S rRNA gene were amplified with the Primer pairs (forward: CCTAYGGGRBGCASCAG, reverse: GGACTACNNGGGTATCTAAT), and sequenced on the Ion Torrent S5XL platform by Novogene (Beijing, China).

### Microbial Data Analysis

The 16S microbial sequencing data were processed and analyzed by Quantitative Insights into Microbial Ecology (QIIME v2 2019.4) (Bolyen et al., 2019). In brief, data firstly were dereplicated using Vsearch, and then clustered to operational taxonomical units (**OTUs**) with a 99% identify cutoff by de novo method (via q2-vsearch). The chimeras and “borderline chimeras” were excluded (via q2-vsearch). For the taxonomic assignment, QIIME-compatible SILVA releases 132 (https://www.arb-silva.de/download/archive/qiime) were used via q2-feature-classifier. The OTUs relative abundance of each sample and 6-level taxonomic classification from phylum to genus was then obtained. The OTUs <0.005% were finally removed from the analysis (via q2-feature-table). Qualified OTUs data were then used to calculate the alpha-diversity of Shannon. And binary Jaccard distance dissimilarities was produced as beta-diversity and then subjected to Principle Coordinate Analysis (**PCoA**).

### Measurement of SCFAs

A total of 40 cecal content (10 repeats for each group, 5 Female /5 Male for each repeat) were randomly selected to test concentration of Short Chain Fatty Acids (SFCAs). About 0.1 to 0.4 g of cecal content was put into 1.5 mL centrifuge tubes and then suspended in Milli-Q water. After centrifugation at 12,000 r/min for 15 min, 0.1 mL of supernatant was mixed with 0.2 mL 25% (w/v) solution of metaphosphoric acid and crotonic acid. Finally, the mixture was used to measure the concentrations of SCFAs (primally including acetic acid, propionic acid, butyric acid, isobutyric acid, valeric acid, and isovaleric acid) by Gas Chromatograph (Varian CP-3800, Palo Alto, CA.).

### Statistical Analysis

The nonparametric Kruskal Wallis was used to test the difference among groups of microbial α diversity. Differentially abundant taxa were identified by linear discriminant analysis (**LDA**) effect size (**LEfSe**). The differences in predicted function outcomes among the groups were compared using the STAMP software v2.1.3 (https://beikolab.cs.dal.ca/software/STAMP). Two-sided Welch's *t* test and Benjamini-Hochberg FDR correction were applied for the two-group analysis. ANOVA with Tukey-Kramer test and Benjamini-Hochberg correction (*P* < 0.05) were utilized for the multiple group analysis. For body weight and SCFAs data, one-way ANOVA with Duncan's test was used at *P* < 0.05 (SPSS 27.0, Chicago, IL).

## RESULTS AND DISCUSSIONS

### Changes of Body Weight and Cecal Microbiota Community With Different Crude Fiber Level in Dahen Broiler

**Figure 1:**
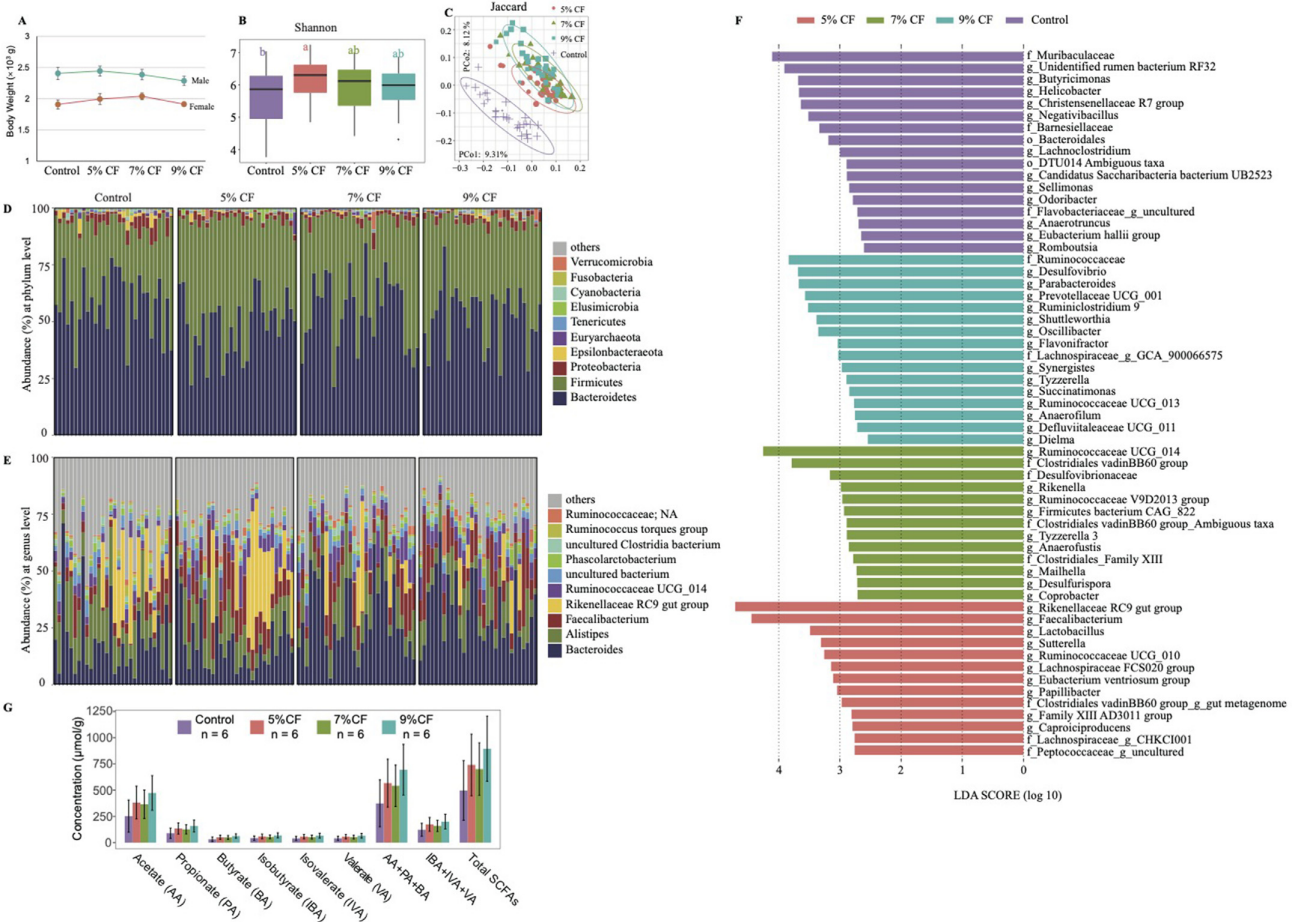
The changes of cecal microbiota with dietary fiber. (A) The body weight in each group at d 75; (B) α-diversity in cecum of Dahen broilers; (C) principal coordinate analysis (PCoA) of microbial community dissimilarity; the composition of gut microbiota of Dahen broiler with different fiber level diet groups at phylum level (D) and genus level (E); (F) microbial taxa significantly different in each group identified by linear discriminant analysis coupled with effect size (LEfSe) using default parameters; (G) concentrations SCFAs in different groups. Abbreviation: SCFAs, short chain fatty acids.

Dietary fiber, to some extent, is not conducive to the nutrient and energy digestibility, but it has potentially beneficial effects on animal intestinal health and welfare. In this study, we found at the end of experiment (d 75), the body weights showed a trend of increasing at 5% CF treatment group compared with control groups (Figure 1A). Previous study also reported that 5 or 6% fiber level in daily diet can significantly increase the growth performance in broiler chickens (Jimenez-Moreno et al., 2016; Taheri and Shirzadegan 2017). The microbial community alpha-diversity also indicated that the dietary crude fiber at 5% CF, 7% CF and 9% CF showed an increasing trend compared with the recommended dietary fiber level (control group with 3% dietary fiber) based on Shannon index (Figure 1B). And the 5% CF group had significant higher community diversity (*P* < 0.05,). Further, we compared the community composition based on Jaccard distance, the results also showed a clear separation of control group from 5, 7, and 9% fiber level (Figure 1C). Some important characteristics have been proposed that increased microbial community richness or diversity or both are related with the host health. So, the different fiber levels may have impact on the final body weight in Dahen Broilers.

### Microbial Community Composition in Different Groups

Next, we tested the changes of microbial composition with the different dietary fiber levels. Consistent with previous studies, *Firmicutes* and *Bacteroidetes* are 2 dominant phyla inhabiting the chicken intestine (Huang et al., 2018). *Proteobacteria, Epsilonbacteraeota, Euryarchaeota, Tenericutes, Elusimicrobia, Cyanobacteria, Fusobacteria*, and *Verrucomicrobia* showed relative low abundance (Figure 1D). At the genus level, the top 10 genera were *Bacteroides, Alistipes, Faecalibacterium, Rikenellaceae* RC9 gut group, and *Ruminococcaceae* UCG 014, *Phascolarctobacterium*, uncultured *Clostridia* bacterium, *Ruminococcus* torques group, and *Ruminococcaceae* group in all groups (Figure 1E). Literature searches provide that the *Bacteroides* have been found it can utilize the fiber polysaccharide (Chijiiwa et al., 2020). *Alistipes* is associated with animal diet, high fat diet or vegetable consumption (David et al., 2014). *Faecalibacterium, Ruminococcaceae, Lactococcaceae* have been found to associate with SCFAs production and the chicken high performance, and family *Rikenellaceae* is involved in degrading carbohydrate (Rowland et al., 2018).

Subsequently, to identify the microbial taxa that were significantly differentiated in each group, especially in different fiber levels, we performed linear discriminant analysis at the genus level in combination with Effect Size (LEfSe) analysis. Figure 1F shows microbial taxa differentially represented in each group. In 5% CF group, 13 specific high abundance biomarkers were identified, such as *Rikenellaceae* RC9 gut group, *Faecalibacterium, Lactobacillus*. 13 microbial taxa were significantly higher in 7% CF (e.g., *Ruminococcaceae* UCG 014, family *Clostridiales* vadinBB60 group, *Desulfovibrionaceae*). And 16 taxa were more abundant in 9% CF group (e.g., *Ruminococcaceae, Desulfovibrio, Parabacteroides, Prevotellaceae* UCG 001, *Ruminiclostridium* 9, *Shuttleworthia, Oscillibacter*). They may involve in amino sugar and nucleotide sugar metabolism, fructose and mannose metabolism, and glycolysis/gluconeogenesis. Together, it suggests that different dietary fiber levels may regulate microbial function.

### SCFA in Different Groups

Gut microbiota in chicken caecum can ferment and degrade the indigestible carbohydrates such as dietary fiber to produce SCFAs as an energy source for the host. The acetic acid, propionic acid and butyric acid are the main metabolic products. Walugembe et al. (2015) found that the proportion of butyrate decreases significantly with increasing dietary fiber content, with less effect on other fatty acids. In this study, no significant differences of the proportions of the 6 SCFAs between groups, although the total SCFAs concentrations were higher in 5%CF, 7%CF, and 9% CF groups compared with control group (Figure 1G). Further correlation analysis showed the relative abundance of the genera *Anaerofilum* and *Dielmawas* was positively correlated with acetate, propionate, butyrate, isobutyrate, isovaleric acid, and valeric acid, which suggest that these 2 genera may involve in the SCFAs production in broiler chickens.

Together, these results provide a reference for the broilers gut microbiota changes with different dietary fiber level, whether and how to the dietary fiber improve chicken growth performance still need more study.
